# Levosimendan versus dobutamine in cardiac surgery

**DOI:** 10.1186/cc14231

**Published:** 2015-03-16

**Authors:** MD Delgado-Amaya, EC Curiel-Balsera, CJ Joya-Montosa

**Affiliations:** 1 Hospital Regional de Málaga, Spain

## Introduction

Early studies suggested a significant increase in survival in patients treated with levosimendan compared with dobutamine or placebo (LIDO, RUSSLAN and CASINO trials). However, two subsequent studies (SURVIVE and REVIVE II) have not confirmed these findings.

## Methods

A prospective observational study of all patients undergoing cardiac surgery at Malaga's Regional Hospital from March 2009 to March 2013. We analyzed patients who used levosimendan compared with those that used dobutamine in the first hours after cardiac surgery, discarding patients in which neither of these two drugs were used or surgical cases that arrived at the ICU with both inotropics. We analyzed demographic variables as well as clinical complications in the ICU and overall perioperative mortality of patients. We performed a second analysis using the propensity score, obtaining the probability of patients being treated with either drug, pairing each patient who received levosimendan with its nearest neighbor receiving dobutamine.

## Results

We collected 875 patients: 331 received one of the two drugs, 50 received both drugs and 494 did not receive any drug. ICU mortality was 7.2% (levosimendan group) and 12.5% (dobutamine group), *P *= 0.1. After adjustment for severity and type of surgery, the use of levosimendan in the postoperative period was not a protective factor for ICU mortality (*P *= 0.18, OR = 0.5, 95% CI = 0.18 to 1.3). In the matched sample, mortality was 7.4% (levosimendan group) and 5.9% (dobutamine), *P *= 0.73. After logistic regression adjusted for severity, measured with EuroSCORE and type of surgery, levosimendan was not a protective factor for ICU mortality (*P *= 0.8, OR = 1.2, 95% CI = 0.26 to 5.45).

## Conclusion

In our environment, we have observed differences in the use of levosimendan compared with dobutamine (higher rate of men undergoing CABG, diabetes and worse EF). After homogenizing the sample of patients by propensity score, an effect on mortality is discarded and we observed a significant need for use of norepinephrine and a nonsignificant trend for prolonged mechanical ventilation and renal failure requiring renal replacement therapy, both probably related with the greatest need for vasopressors observed.

